# Case Report: Pink Urine Syndrome Following Exposure to Propofol: A Rare, Impressive but Benign Complication

**DOI:** 10.3389/fphar.2021.686619

**Published:** 2021-06-15

**Authors:** Fangwei Zhang, Xing Zhu, Hongbo Zhang, Lin Xu, Weiguo Wu, Xuelei Hu, Haipeng Zhou, Penghui Wei, Jianjun Li

**Affiliations:** ^1^ Department of Anesthesiology, Qilu Hospital (Qingdao), Cheeloo College of Medicine, Shandong University, Qingdao, China; ^2^ Department of Pathology, Qilu Hospital (Qingdao), Cheeloo College of Medicine, Shandong University, Qingdao, China; ^3^ Department of Clinical Laboratory, Qilu Hospital (Qingdao), Cheeloo College of Medicine, Shandong University, Qingdao, China; ^4^ Department of Thoracic Surgery, Qilu Hospital (Qingdao), Cheeloo College of Medicine, Shandong University, Qingdao, China

**Keywords:** propofol, pink urine syndrome, uric acid metabolism disorders, non-obese patients, complications

## Abstract

Drug-induced changes in urine color induced by drugs may have clinical significance. Pink urine syndrome (PUS), which has been associated with urinary uric acid (UA) disorders, is most frequently reported in patients with morbid obesity undergoing gastric bypass surgery and/or from propofol anesthesia use in those who potentially have preexisting UA metabolism disorders. However, PUS has rarely occurred following exposure to propofol in non-obese patients, and literature on long-term follow-up after PUS is scarce. We report a case of PUS induced by propofol in a previously healthy non-obese woman after undergoing thoracoscopic wedge resection of pulmonary nodules under general anesthesia using propofol. The patient suddenly developed pink urine 4 h after surgery. A pink sediment rapidly precipitated at the bottom of the test tube following centrifugation of the urine. Amorphous, colorless UA-like crystals were identified under a polarizing microscope. The diagnosis of PUS was confirmed by examining the urinary UA concentration. The patient recovered and as followed-up for 1 month, during which she did not experience any urinary complications. To our knowledge, this is the first report to describe in detail a case of PUS caused by propofol in a non-obese patient with follow-up. PUS is usually benign and can resolve by rapidly on administering lactated Ringer’s solution; however, the potential risk of urinary complications, particularly UA lithiasis, should be fully realized.

## Introduction

Urine discoloration caused by chemicals or drugs is an important clinical symptom that indicates a potentially dangerous condition, which may also be disturbing for patients and clinicians (Chindarkar et al., 2014). Propofol (2,6-diisopropylphenol) is a commonly used intravenous anesthetic and sedative agent administered during surgery and in intensive care units (Wang et al., 2019). It is widely used as a first-line agent to sedate intubated and mechanically ventilated patients with coronavirus disease 2019 (COVID-19) and acute respiratory distress syndrome occurring during the COVID-19 pandemic (Wei et al., 2021). Among anesthetic medications, propofol has most frequently been reported as a risk factor for urine discoloration (Tucker and Perazella, 2019). White and green coloration of urine in patients receiving propofol treatment has been described in detail (Tucker and Perazella, 2019). White urine is caused by the oil-in-water emulsion vehicle of propofol (Nates et al., 1995); green urine, reported in three leading medical journals (New England Journal of Medicine, The Lancet and Journal of the American Medical Association), is attributed to the phenolic metabolites of propofol, which are produced by the liver and eliminated through the kidneys (Norfleet, 1982; Leclercq et al., 2009; Boshkovska Spaseski and Spaseski, 2020). The green and white colors of urine due to propofol are benign and self-limiting. Although they have no clinical significance, earlier recognition of these complications may avoid unnecessary laboratory monitoring (Blakey and Hixson-Wallace, 2000; Barbara et al., 2012). Pink urine due to propofol treatment has rarely been reported, particularly in non-obese patients. We report a case of pink urine syndrome (PUS) that occurred in association with propofol exposure during general anesthesia in a non-obese woman.

## Case Presentation

**FIGURE 1:**
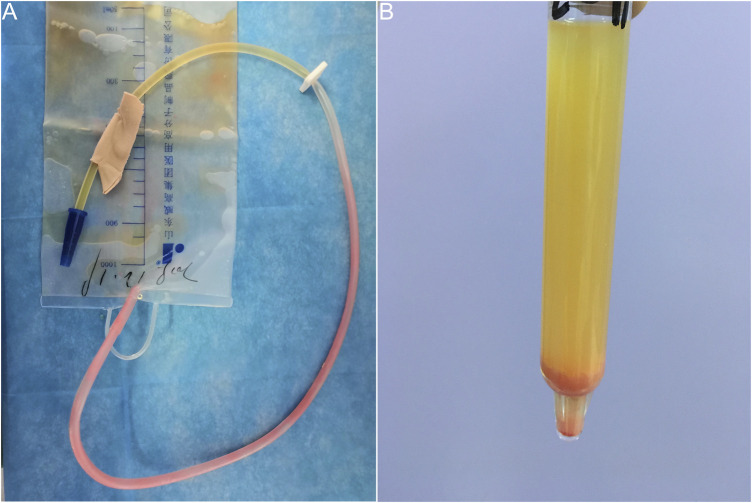
Pink urine syndrome associated with propofol use during general anesthesia. **(A)** Pink urine in the urinary drainage bag and numerous pink substances on walls of the catheter. **(B)** Pink sediment rapidly precipitated in the urine following centrifugation.

A 41-year-old woman with suddenly excreted pink urine after undergoing thoracoscopic wedge resection of pulmonary nodules under general anesthesia. Her medical history otherwise was not significant. She reported that the pink substance was initially seen on the walls of the indwelling urethral catheter 4 h post-operatively. It gradually increased and ultimately stabilized. She was otherwise well and experienced no significant urinary irritation. The following morning, we observed pink urine in the urinary drainage bag and numerous pink crystals on the walls of the catheter (Figure 1A). The patient had no history of gout, diabetes, or renal disease. Notably, no urinary abnormalities were observed when she had previously undergone urinary catheterization after spinal anesthesia for a cesarean section and received postoperative intravenous patient-controlled analgesia in our hospital.

On examination, the patient had a body mass index of 25.71 kg m^−2^ and her temperature was normal. Renal percussion revealed no pain and no other abnormalities were found. Preoperative laboratory investigations showed a serum uric acid (UA) concentration of 218 μmol L^−1^ (normal 150–350), morning urinary pH of 6.5 (normal 4.5–8.0) and UA concentration of 3,320 μmol L^−1^ (normal 2,200–5,475). On postoperative day 1, the serum UA concentration was 166 μmol L^−1^, morning urinary UA concentration was 4,825 μmol L^−1^ and urinalysis revealed a pH of 6. Preoperative and postoperative urinalyses and renal function tests showed no other abnormalities.

**FIGURE 2:**
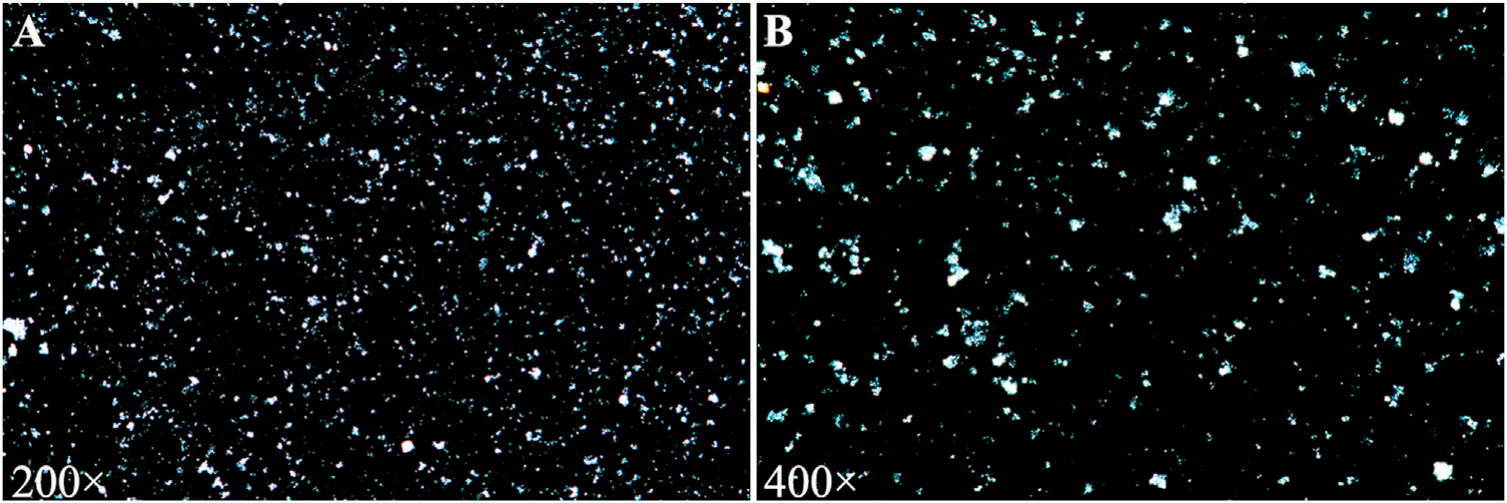
Urine sediment examination showing amorphous and colorless uric acid crystals under a polarizing microscope [**(A)** 200×; **(B)** 400×].

**FIGURE 3:**
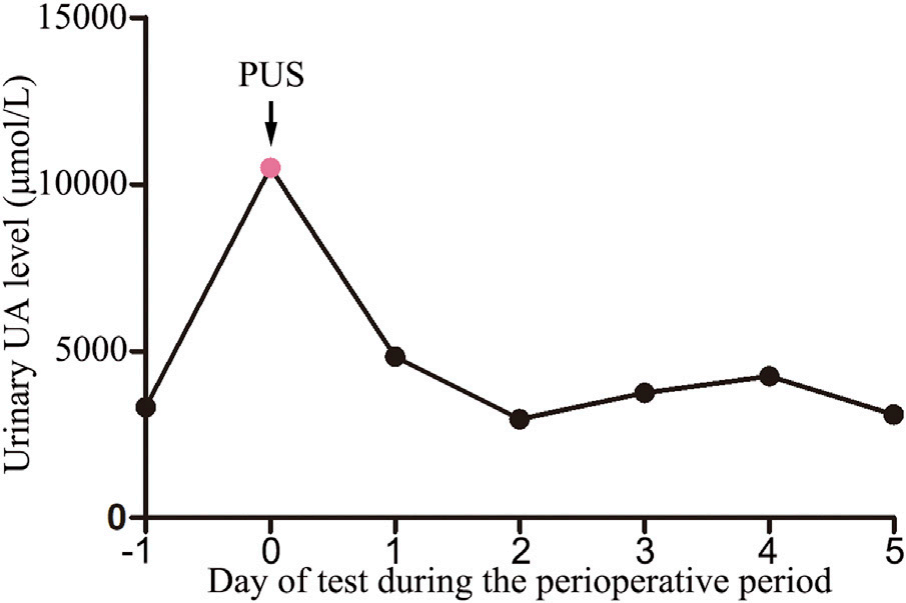
Urinary uric acid levels during the perioperative period (−1: before surgery; 0: urinary drainage bag; 1–5: days after surgery). PUS: Pink Urine Syndrome.

A urinalysis using samples from the urinary drainage bag did not reveal red cells or free hemoglobin. The urine cultures were sterile. A pink sediment rapidly precipitated in the urine following centrifugation (Figure 1B). Under a polarizing microscope, amorphous, colorless UA-like crystals were identified (Figure 2). We further examined the urinary UA concentration and the pH value and found that it was significantly increased in UA concentration (10,490 μmol L^−1^, lower than the real value due to UA precipitation on the catheter walls) and decreased in pH (5) (Figure 3).

The patient had received no premedication, and general anesthesia had been performed using propofol, dexmedetomidine, sufentanil, cisatracurium, sevoflurane, and remifentanil. Postoperative analgesia was achieved using oxycodone, sufentanil and parecoxib sodium. The patient did not receive any other medications or treatment up to the point of excretion of pink urine, apart from the routine fluid therapy. The concentration of UA in the patient’s morning urine, 4 days post-operatively, was within normal limits (Figure 3). The patient was followed up for 1 month and did not experience any urinary complications.

## Discussion

PUS is a rare condition with uric acid metabolism disorders characterized by strawberry-red urine and the formation of massive pink sediment (Potton et al., 2013). Pure UA dihydrate crystals are amorphous and colorless but can become pink upon absorbing pink urinary pigments (Tan and Lai, 2012). PUS has been most frequently observed in patients with morbid obesity and/or insulin resistance, which may potentially involve UA metabolic disorders (Deitel et al., 1984; Tucker and Perazella, 2019). Obesity reportedly promotes purine biosynthesis and UA production, with consequent hyperuricosuria contributing to the acidification of urine, which facilitates UA secretion into the urine (Negri et al., 2008). Studies showed that higher the potential baseline level of UA in the urine, greater is the possibility of patients with obesity undergoing gastric partitioning (Deitel et al., 1984). Insulin resistance can increase serum UA concentration by reducing renal clearance and UA excretion (Perez-Ruiz et al., 2015). Furthermore, there is a strong correlation between insulin resistance and a low urinary pH, which is a necessary factor for UA crystallization (Maalouf et al., 2007). However, our patient had no clinical history or evidence of preexisting UA metabolic disorders.

**FIGURE 4:**
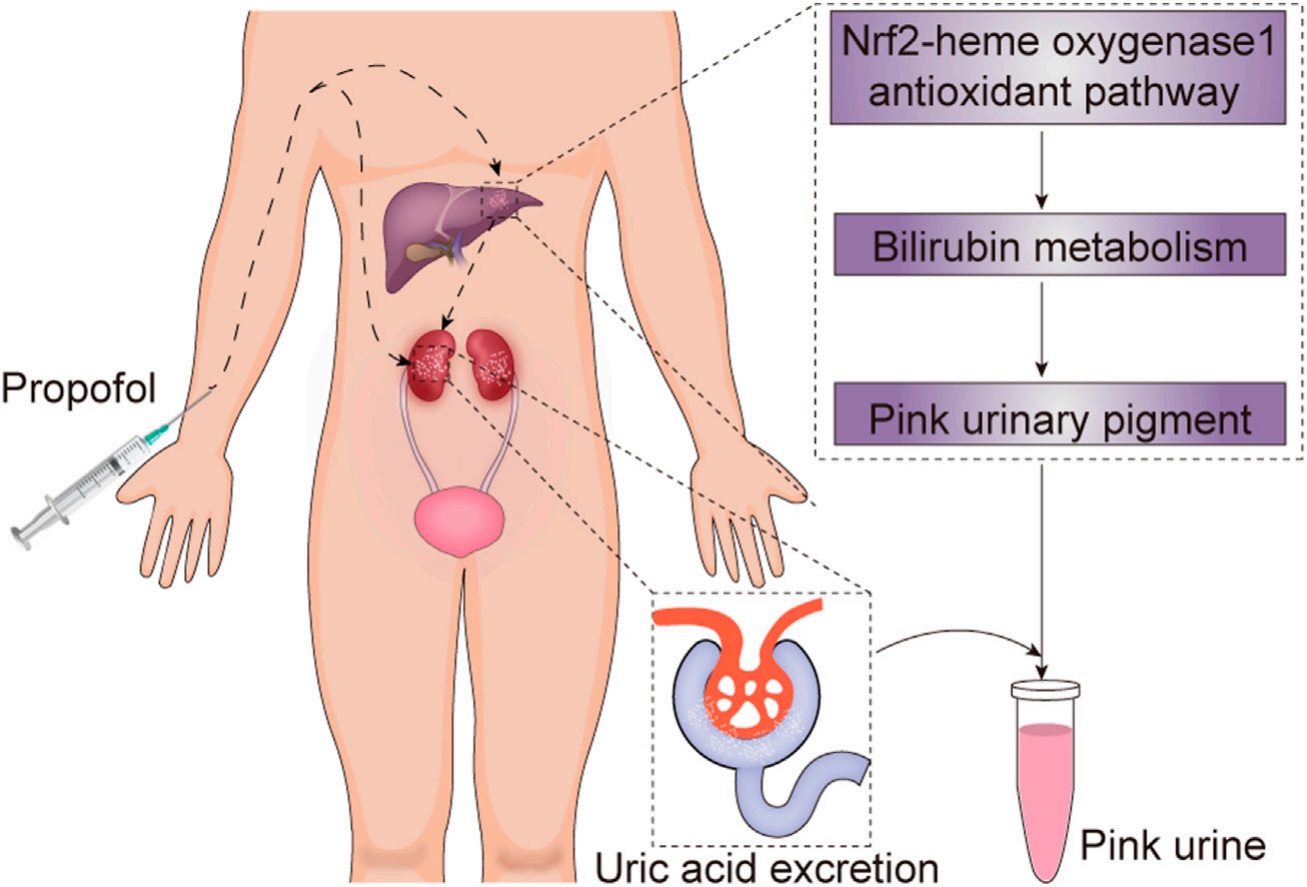
The potential mechanism underlying propofol-induced pink urine syndrome. Propofol can directly increase colorless uric acid excretion and promote pink urinary pigment production through bilirubin metabolism by activating the Nrf2-heme oxygenase-1 antioxidant pathway, and consequently result in pink urine syndrome.

Another major cause of PUS is propofol. Propofol, a dialkylphenol, is a gamma-aminobutyric acid (GABA) receptor agonist. It can potentiate the response to the inhibitory neurotransmitter GABA at the GABA_A_ receptor, and cause sedative and hypnotic effects (Brohan and Goudra, 2017). Propofol is metabolized mostly by the liver to various inactive sulfate and glucuronide metabolites (Sahinovic et al., 2018). Propofol infusions have been found to be closely associated with high urinary UA excretion and pink urine discoloration, though PUS has rarely been described in non-obese patients receiving propofol treatment (Masuda et al., 1997; Stern et al., 2010). The exact mechanisms underlying propofol-induced PUS remain unclear. A study conducted by Masuda et al. (1997) demonstrated that propofol infusions can directly increase colorless urate excretion in the urine; the results showed that propofol cleared significantly more urate (mean 22.9 ml/min) than sevoflurane (mean 5.9 ml/min) in patients with an American Society of Anesthesiologists physical status I or II. Interestingly, no significant difference in urinary pH, creatinine clearance, or urine volume between propofol and sevoflurane were found. The mechanisms remain to be explored. Additionally, recent evidence has shown that propofol plays an indirect role in PUS. The mechanism may involve activation of the Nrf2-heme oxygenase-1 antioxidant pathway, which promotes pink urinary pigment production through bilirubin metabolism (Masuda et al., 1997; Tucker and Perazella, 2019). Therefore, the combination of direct and indirect effects of propofol on UA metabolism may consequently result in pink urine. Considering the patient’s presentation, we believe that PUS in this case was secondary to propofol administration. The potential mechanisms by which propofol treatment leads to PUS in non-patients are summarized (Figure 4).

Other risk factors, including V1 receptor activation, low urinary pH and high urine osmolarity, have been implicated in PUS (Tan and Lai, 2012; Tucker and Perazella, 2019). Stress-related antidiuretic hormone release, *via* its V1 receptor, favors renal UA clearance and causes UA crystalluria (Tan and Lai, 2012). A low urinary pH attributed to intraoperative potential respiratory acidosis and high urine osmolarity associated with dehydration may also be involved in PUS (Tucker and Perazella, 2019). The reduction in the excretion of urinary glutamate may also be responsible for the lowering of urinary pH in PUS (Ogawa et al., 2016). Although V1 receptor activation mediated by surgical stress and decreased urinary pH may be involved in the development of PUS, a stronger factor in its development is propofol (Tucker and Perazella, 2019).

To our knowledge, this is the first case report to describe in detail the development of PUS induced by propofol with follow-up in a non-obese patient. Understanding the nature of PUS allays distress among patients and clinicians, prevents unnecessary testing such as urinalysis, urine cultures and blood cultures, and enables prompt treatment (Blakey and Hixson-Wallace, 2000). Clinically, PUS has a quick onset and can be rapidly resolved by administering lactated Ringer’s solution. PUS is usually benign, but the potential risk of urinary complications like UA lithiasis should be fully realized (Potton et al., 2013).

## Conclusion

In conclusion, PUS can occur in non-obese patients with no preexisting UA metabolic disorders. Propofol is a key risk factor for PUS in such patients, which directly increases colorless UA excretion in urine and promotes pink urinary pigment production through bilirubin metabolism. High urinary excretion of UA crystals may be transient with no toxicity to the human body. Therefore, unnecessary testing should be avoided. Despite its benign nature, prompt intravenous fluid therapy is still necessary to prevent urinary complications.

## Data Availability

The original contributions presented in the study are included in the article/Supplementary Material, further inquiries can be directed to the corresponding authors.
